# Resistance to everolimus driven by epigenetic regulation of MYC in ER+ breast cancers

**DOI:** 10.18632/oncotarget.2964

**Published:** 2014-12-11

**Authors:** Teeru Bihani, Scott A. Ezell, Brendon Ladd, Shaun E. Grosskurth, Anne Marie Mazzola, Mark Pietras, Corinne Reimer, Michael Zinda, Stephen Fawell, Celina M. D'Cruz

**Affiliations:** ^1^ AstraZeneca, R&D Boston, Waltham, MA

**Keywords:** everolimus, resistance, mTOR, MYC, BRD4

## Abstract

Acquired resistance to PI3K/mTOR/Akt pathway inhibitors is often associated with compensatory feedback loops involving the activation of oncogenes. Here, we have generated everolimus resistance in ER+ breast cancer cells and in long-term estrogen deprived (LTED) models that mimic progression on anti-estrogens. This allowed us to uncover MYC as a driver of mTOR inhibitor resistance. We demonstrate that both everolimus resistance and acute treatment of everolimus can lead to the upregulation of MYC mRNA, protein expression and, consequently, the enrichment of MYC signatures as revealed by RNA sequencing data. Depletion of *MYC* resulted in resensitization to everolimus, confirming its functional importance in this setting. Furthermore, ChIP assays demonstrate that *MYC* upregulation in the everolimus resistant lines is mediated by increased association of the BRD4 transcription factor with the *MYC* gene. Finally, JQ1, a BRD4 inhibitor combined with everolimus exhibited increased tumor growth inhibition in 3D Matrigel models and an in vivo xenograft model. These data suggest that MYC plays an important role in mediating resistance to everolimus in ER+ and ER+/LTED models. Furthermore, given the regulation of*MYC*by BRD4 in this setting, these data have implications for increased therapeutic potential of combining epigenetic agents with mTOR inhibitors to effectively downregulate otherwise difficult to target transcription factors such as MYC.

## INTRODUCTION

Breast cancer is the most frequent type of cancer diagnosed and is responsible for the second most fatalities in women. An extremely heterogeneous disease, breast cancer is comprised of patients that fall into different disease segments based on their tumor's histotype; namely, hormone (estrogen and progesterone receptors) and Her2 status. The largest disease segment within breast cancer is hormone receptor positive disease, with approximately 75% of patients falling into this category [1-3].

Estrogen receptor-positive (ER+) tumors typically rely on circulating estrogen for their growth. Given this dependence, treatment options for ER+ breast cancer patients have relied heavily on anti-hormonal strategies with varying anti-estrogen modalities. These include such agents as tamoxifen (competes with estrogen for binding to ER) [4], aromatase inhibitors (prevents biosynthesis of estrogen) [5-7] and fulvestrant (downregulates ER) [8], all of which have shown success in the clinic. More specifically, third generation aromatase inhibitors (letrozole, anastrazole and exemestane) have shown improved overall survival and have become the standard of care for postmenopausal women with ER+ breast cancer [5-7]. While most patients respond to endocrine agents, eventually a majority will display resistance to such agents [9]. Understanding the molecular mechanisms that drive this resistance is crucial to overcoming these clinical hurdles. It is necessary, therefore, to have appropriate biological models in place to help determine molecular drivers of resistance and to test pre-clinical hypotheses.

The long-term estrogen deprived (LTED) model originally developed by the Arteaga group involves growing ER+ breast cancer lines in the absence of estrogen [10]. These LTED cell lines eventually lose their dependence on estrogen and mimic clinical tumors progressing on tamoxifen or aromatase inhibitors [10]. Their predictive nature is underscored by the similarities between LTED gene signatures and the gene signatures of patients treated with hormonal agents. These LTED models provide very useful tools in understanding primary and secondary mechanisms of resistance in ER+ breast cancer patients. Indeed, the LTED model has implicated the PI3K/Akt/mTOR pathway's involvement in mediating resistance to anti-estrogens [10]. These and other studies have resulted in many initiatives to understand and target members of the PI3K/Akt/mTOR family.

Everolimus (Rad001), is an allosteric inhibitor of mTOR complex 1 (mTORC1), demonstrating efficacy in many different cancer types, including renal cell carcinoma, pancreatic neuroendocrine tumors and advanced kidney tumors, all of which everolimus is FDA-approved for [11]. Most recently, FDA approval was achieved based on data from the BOLERO-2 trial, demonstrating that everolimus in conjunction with exemestane, an aromatase inhibitor, improves progression-free survival compared to exemestane alone in post-menopausal women with advanced ER+, Her2-negative breast cancer [12-13]. Very importantly, while progression-free survival was significantly improved, recent data has been released suggesting no improved overall survival in this disease segment [14]. These recent data suggest that the combination of exemestane and everolimus does not give a durable clinical response, indicating a need for alternative combinations and therapeutic strategies.

To understand and overcome this potential onset of drug resistance, here we describe the generation of everolimus-resistant cell lines both in parental and long-term estrogen-deprived (LTED) backgrounds of ER+ breast cancer cell lines. We demonstrate that cells resistant to everolimus have an activated MYC signature due to upregulation of MYC expression at the transcript and protein levels. We further show that this upregulation of MYC is mediated by BRD4 regulation of the *MYC* gene and that combining a BRD4 inhibitor with everolimus leads to enhanced tumor growth inhibition *in vitro* and *in vivo*.

## RESULTS

### The generation and validation of everolimus resistance in parental and LTED ER+ breast cancer cell lines

**Figure 1:**
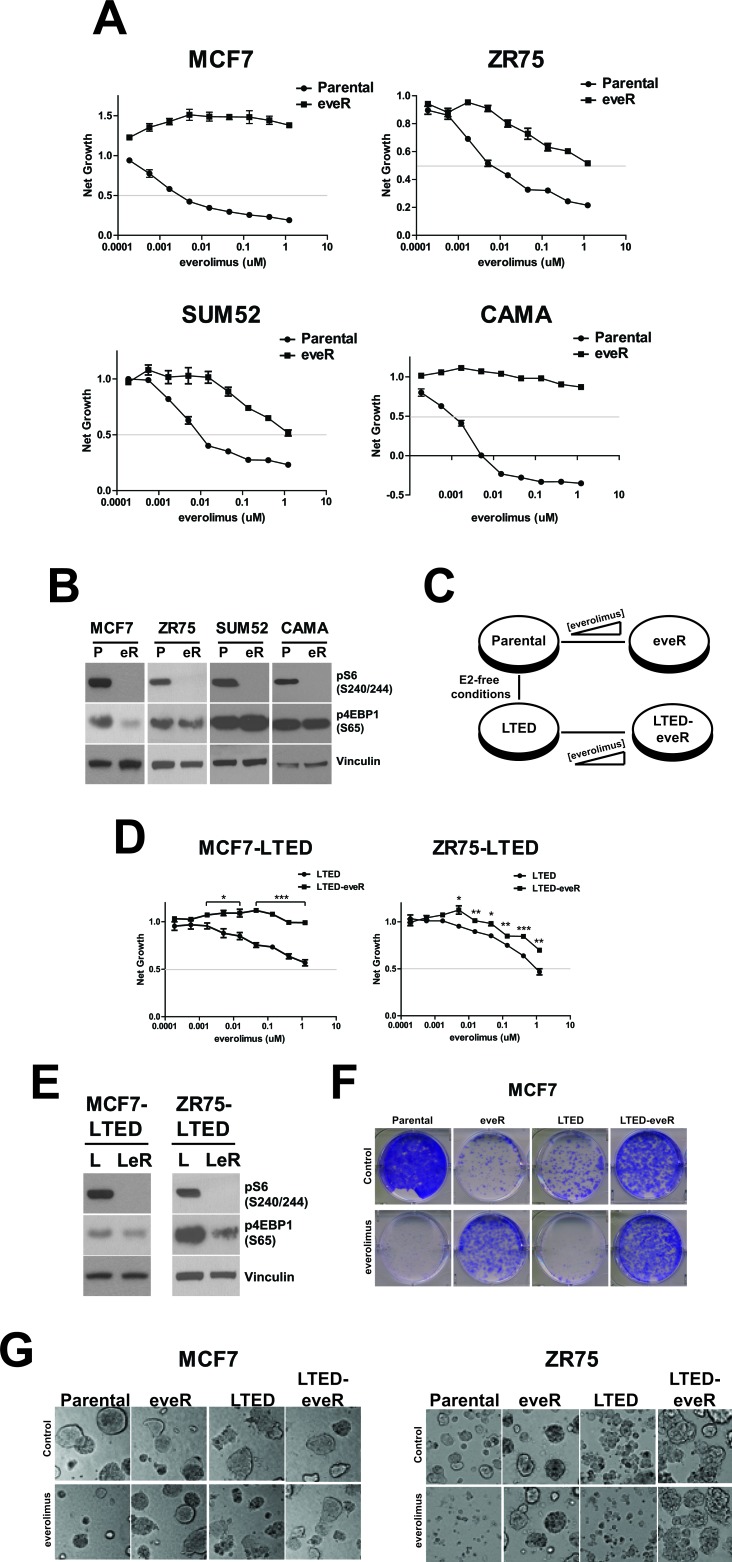
Resistance to everolimus in ER+ cell lines A. The indicated cell lines were incubated for five days in the presence of everolimus before measurement of proliferation using CellTiterGlo. Points depict the average net growth. Bars indicate SEM. B. Western blot analyses of indicated cell lines. Representative samples were immunoblotted for the indicated proteins. α-Vinculin was used as a loading control. P: Parental, eR: eveR. C. Diagram depicting the generation of everolimus resistance in parental and LTED backgrounds. E2: estrogen. D. Proliferation of LTED derivatives as in A. E. Western blot analyses of LTED derivatives as in B. F. Colony formation of the indicated cell lines. Cells were plated in the presence of 500nM everolimus or DMSO solvent control. Representative images are shown. G. MCF7 and ZR75 derivative cell lines grown in three-dimensional Matrigel culture for five days in the presence everolimus (500nM) or DMSO. Representative images were taken five days post-treatment using a 20X objective.

We chose four ER+ breast cancer cell lines to study mechanisms of everolimus resistance; MCF7, ZR75-1 (ZR75), SUM52 and CAMA-1 (CAMA). To generate resistant models (eveR), cells were exposed to increasing concentrations of everolimus until growth inhibition in the presence of compound was no greater than 50% (and the GI50 was significantly different than parental counterparts) (Figure 1A). Importantly, parental lines were maintained in culture for similar lengths of time as the everolimus-resistant versions, to control for long-term culture effects.

We next assessed biochemical changes in the eveR lines. Acute inhibition of mTOR by everolimus should result in lack of kinase activity and consequently, decreased phospho-targets specific to mTOR (e.g. S6 at Serine 240/244 (pS6) and 4EBP1 at Serine 65 (p4EBP1). To determine whether alterations in the mTOR pathway were responsible for the onset of resistance, pS6 and p4EBP1 expression was examined. Importantly, pS6, an mTOR-p70S6K target, was relatively undetectable levels in all eveR lines after extended exposure to everolimus, suggesting this portion of mTORC1 signaling remains targetable in this context and is likely not contributing to the resistant phenotype (Figure 1B). In contrast, we observed a variable response in p4EBP1. Phosphorylation of 4EBP1 was decreased in MCF7-eveR cells as well as in ZR75-eveR, albeit to a lesser extent. In contrast, p4EBP1 was slightly upregulated in SUM52 cells, and was relatively unchanged in CAMA-eveR cells (Figure 1B). In addition, western blot analyses of ER expression was assessed in the eveR derivatives. While eveR in MCF7 resulted in decreased ER expression, the opposite result was seen in CAMA and ZR75 cells (Supplemental Figure 1). Although pS6 is consistently downregulated in eveR lines, the variability in 4EBP1 phosphorylation and ER expression levels suggests context-dependent responses to long-term everolimus treatment.

Given clinical treatment regimens, we hypothesized that generating everolimus resistance in cell lines that model aromatase inhibitor treatment would parallel that of treatment orders seen in patients. Indeed, patients previously treated with endocrine agents were the focus of the BOLERO-2 trial, and for which everolimus treatment has been approved despite lack of change in overall survival in the clinical study [12-14]. For this reason, in addition to generating resistance to everolimus in the four ER+ parental breast cancer cell lines, we also generated resistance in MCF7 and ZR75 LTED counterparts. This resulted in four derivatives of MCF7 and ZR75 cells: long-term culture (Parental, Par, P), everolimus-resistant (eveR, eR), long-term estrogen deprived (LTED, L) and everolimus-resistant LTED (LTED-eveR, LeR) (Figure 1C). In a short-term cell proliferation assay, MCF7-LTED and ZR75-LTED were much less sensitive to everolimus than their parental counterparts (Figure 1A and 1D). Despite this, long-term exposure to everolimus resulted in complete resistance to the drug in the MCF7 setting (Figure 1D). While ZR75-LTED exhibited relative resistance to everolimus compared to the parental cells, ZR75-LTED-eveR exhibited significantly increased resistance to the drug (Figure 1D). Downstream targets of mTOR signaling were assessed in LTED derivatives as well. pS6 was undetectable in both LTED-eveR lines similar to eveR lines. p4EBP1 was decreased in both LTED-eveR, however only to a modest extent in the MCF7-LTED-eveR setting (Figure 1E). Furthermore, LTED derivatives of MCF7 and ZR75 both resulted in significant loss of ER expression (Supplemental Figure 1). The ER downregulation in the LTED lines is not surprising, as it has been previously shown that anti-estrogens can have varying effects on ER levels in patients [15].

To rule out differences in growth rates between derivatives, cells were also plated in a long-term colony formation assay (Figure 1F). The ability of MCF7 parental cells to form colonies in the presence of everolimus was severely impaired. In contrast, MCF7-eveR cells were not only able to form colonies in the presence of everolimus, they did so to a greater extent than in the absence of drug. As expected, the MCF7-LTED line maintained sensitivity to everolimus in the colony-formation assay, and the MCF7-LTED-eveR derivative was able to grow in the presence and absence of drug (Figure 1F). In fact, the MCF7-LTED-eveR cells formed colonies better than the MCF7-LTED regardless of everolimus treatment. These data suggest that lack of sensitivity to everolimus in the eveR and LTED-eveR lines was not due to any differences in growth rates between derivatives. In addition, washout studies demonstrated that eveR and LTED-eveR lines maintained their resistance to everolimus up to at least seven days after washing the cells out of drug (data not shown). To determine whether anchorage-independence and/or contact with extracellular matrix proteins could alter the resistant derivatives' sensitivity to everolimus, growth was assessed in a three-dimensional Matrigel assay [16]. The eveR and LTED-eveR cells remained resistant to everolimus in 3D Matrigel cultures (Figure 1G), suggesting the cells attachment to basement membrane did not alter sensitivity. Interestingly, ZR75-LTED appeared to be more sensitive to everolimus in 3D relative to the 2D assay. In either case, ZR75-LTED-eveR was significantly resistant to everolimus compared to ZR75-LTED cells regardless of assay type (Figure 1D and 1G).

These data demonstrate the generation of cell lines that are resistant to everolimus treatment in a variety of proliferation and growth assays and were considered relevant model systems for further molecular characterization to determine drivers of resistance. Potent inhibition of pS6 in all eveR lines suggests that feedback loops are not playing a role in reactivating pS6, and thus this portion of mTOR signaling is likely not driving resistance. Furthermore, the variable responses to another mTOR target, p4EBP1 prompted us to investigate whether additional players outside of direct mTOR signaling could play a more dependable role in all of the eveR lines.

### RNAseq analyses reveal MYC signatures in eveR and LTED-eveR lines

**Figure 2:**
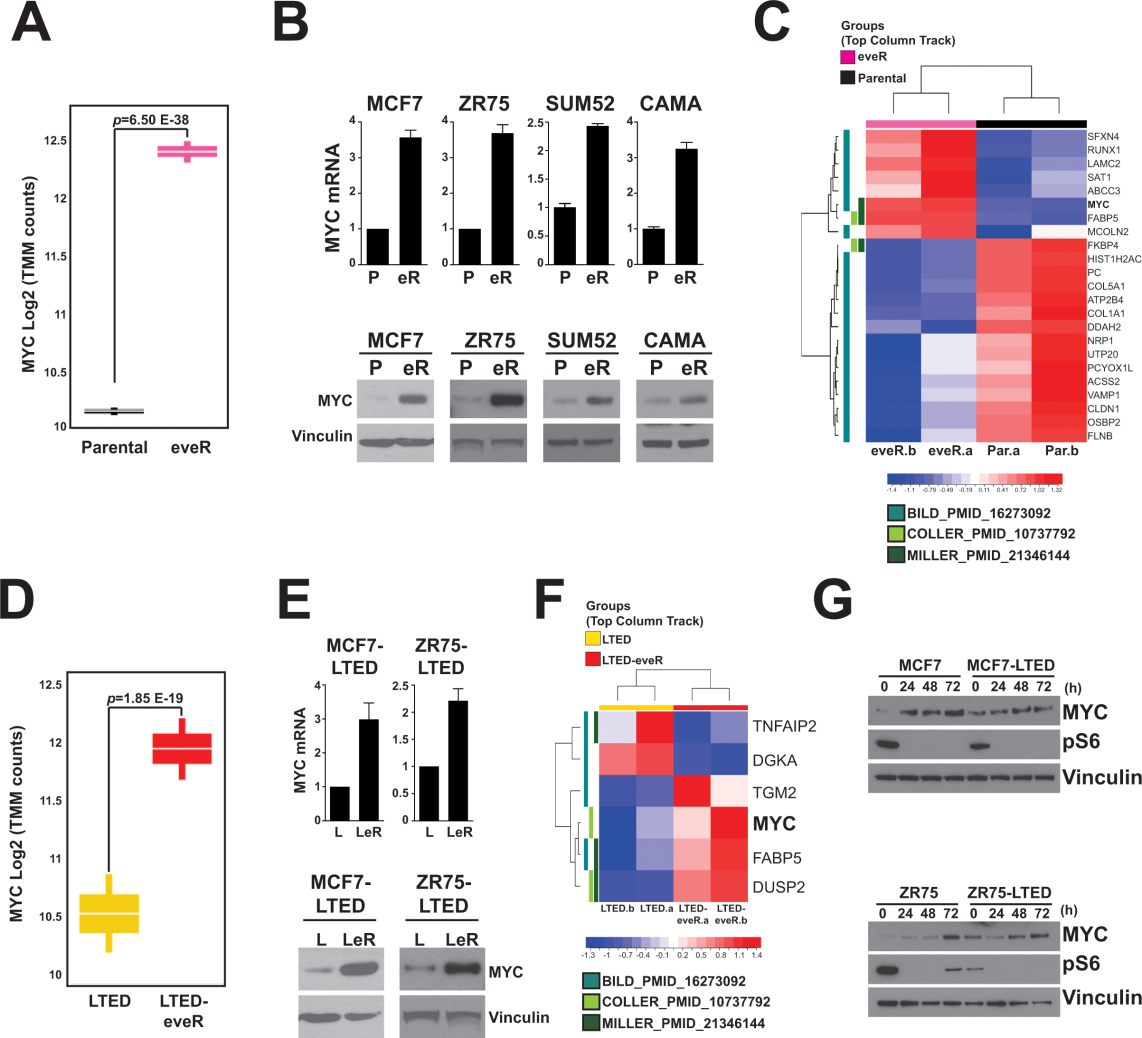
Enrichment of MYC signatures in eveR and LTED-eveR lines A. Differential mRNA expression analyses of MCF7 parental and eveR lines. Two biological replicates of each sample were sent for RNA sequencing. Graph represents the log2 TMM MYC counts. B. (top) Real-time PCR analyses measuring relative MYC mRNA expression in the indicated lines (P: Parental, eR: eveR). Data is represented as fold change over parental. Bars indicate SEM. (bottom) MYC protein expression by western blot analyses in the indicated cell lines. eveR lines were maintained in 500nM everolimus and media was replaced with fresh compound 16-24h prior to harvesting. α-Vinculin was used as a loading control. C. Normalized Counts for 23 Statistically Differentially Expressed MYC Regulated Genes represented as Z-Score Log2 TMM in eveR cells versus Parental cells. D. Differential mRNA expression analyses of MCF7-LTED and LTED-eveR lines. Two biological replicates of each sample were sent for RNA sequencing. Graph represents the log2 TMM MYC counts. E. (top) Real-time PCR analyses measuring relative MYC mRNA expression in the indicated lines (L: LTED, LeR: LTED-eveR). Data is represented as fold change over LTED controls. Bars indicate SEM. (bottom) MYC protein expression by western blot analyses in the indicated cell lines. LTED-eveR lines were maintained in 500nM everolimus and media was replaced with fresh compound 16-24h prior to harvesting α-Vinculin was used as a loading control. F. Normalized Counts for 6 Statistically Differentially Expressed MYC Regulated Genes represented as Z-Score Log2 TMM in LTED-eveR cells versus LTED cells. G. MYC protein levels measured by western blot analyses of parental and LTED derivatives treated with 500nM everolimus for the indicated timepoints (h: hours post-treatment). pS6: α-pS6 (S240/244). α-Vinculin was used as a loading control.

To determine molecular changes that might contribute to the resistance to everolimus, we performed whole transcriptome RNA sequencing to provide a global view of altered pathways in MCF7 derivatives. One of the most robust changes observed was an increase in MYC mRNA expression in the MCF7-eveR line compared to the MCF7 parental line (Figure 2A). Using quantitative real-time PCR analyses, we validated the RNA sequencing result demonstrating that *MYC* mRNA is increased in all of the eveR lines (Figure 2B, top). Furthermore, increased protein expression of MYC was also seen in all eveR lines (Figure 2B, bottom). Gene Set Enrichment Analyses (GSEA) was used to identify gene signatures associated with resistance and in concordance with increased MYC expression, multiple MYC signatures were enriched (Supplemental Table 1), including those previously identified in LTED experiments [17]. Additionally, we show breast cancer specific MYC genes that are statistically differentially expressed between MCF7-eveR and MCF7-parental, suggesting the increased MYC is functional in this setting (Figure 2C) [17-19].

Similar to the parental and eveR lines, we saw an increase in MYC mRNA by RNA sequencing in the MCF7-LTED-eveR line compared to the MCF7-LTED line (Figure 2D). These results were validated by qPCR and western blot analyses in both the MCF7 and ZR75 context (Figure 2E). Furthermore, the LTED-eveR also showed activation of MYC signatures, including breast-specific MYC target genes (Figure 2F and Supplemental Table 1). These data suggest a common mechanism of MYC upregulation and activation in response to everolimus among ER+ breast cancer lines and their LTED counterparts.

Finally, to rule out the upregulation of MYC being due to clonal selection or long-term drug treatment effects, we also examined the expression of MYC in response to acute treatments of everolimus. Similar to the resistant setting, acute treatment of everolimus resulted in an upregulation of MYC protein in both parental and LTED derivatives of MCF7 and ZR75 cells within 72 hours post-treatment (Figure 2G). This suggests that a mechanism to upregulate MYC expression exists in response to treatment of everolimus and targeting MYC and mTOR together might be an effective therapeutic strategy in preventing the onset of everolimus resistance.

### MYC is a driver of resistance to everolimus

**Figure 3:**
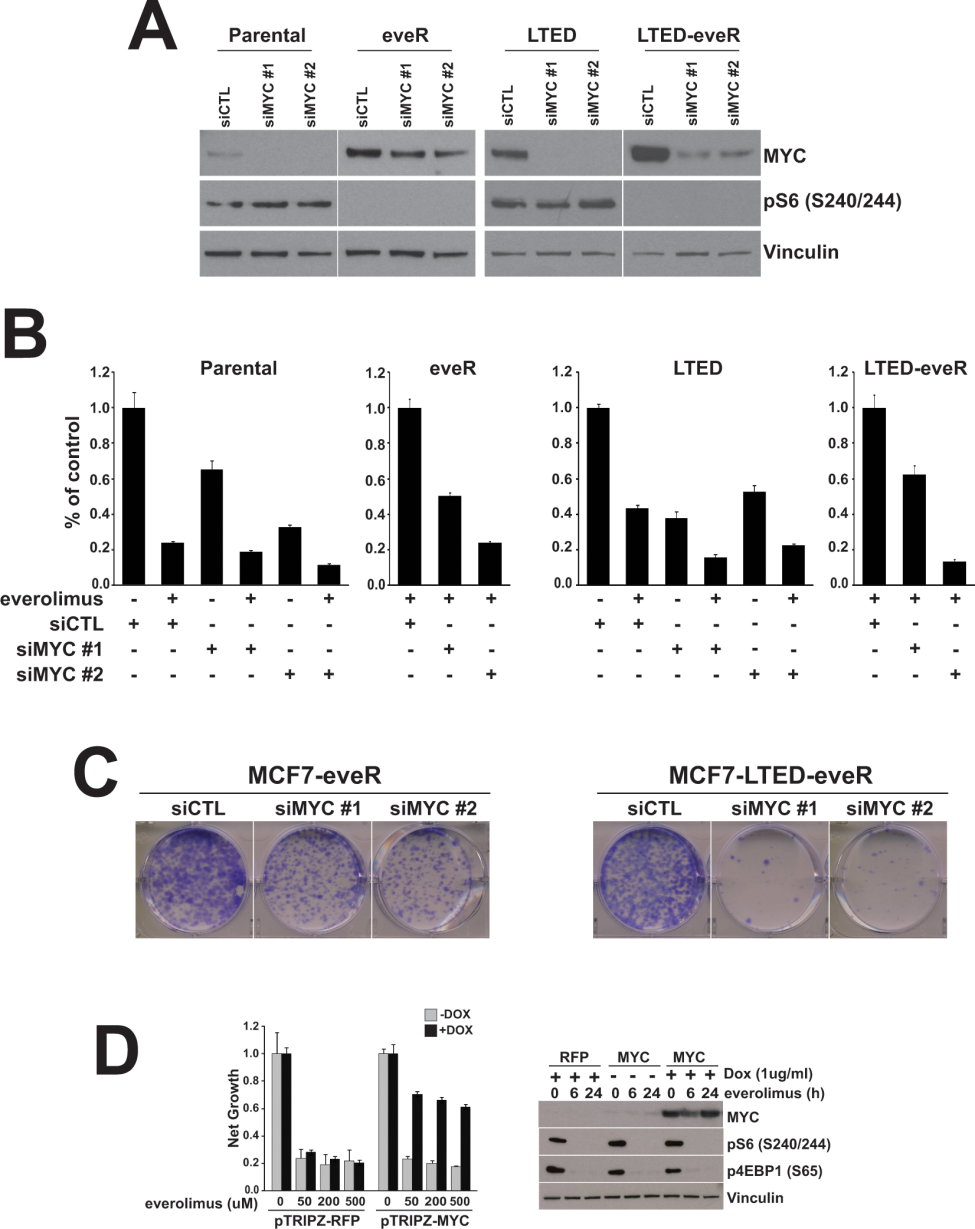
MYC is necessary and sufficient to confer resistance to everolimus A. MYC protein levels measured by western blot analyses of MCF7 derivatives transfected with indicated siRNAs. The eveR and LTED-eveR lines were treated with everolimus (500nM) post-transfection and prior to harvesting. α-Vinculin was used as a loading control. B. Proliferation assay of same cells as in A (+/− everolimus, 500nM) and four days post-transfection. Percent of control with standard deviation is depicted in the histograms. C. Colony formation of the indicated cell lines. Cells were plated in the presence of 500nM everolimus. Images of representative wells are shown. D. MCF7 cells expressing pTRIPZ-RFP or pTRIP-MYC were plated in the presence or absence of 1ug/ml doxycycline (DOX). (left) One day post-plating cells were treated as indicated for four days before lysis with CellTiterGlo reagent. Net growth with standard deviation is depicted in the histograms. (right) Western blot analyses of the indicated proteins. Treatment with doxycycline 24 hours prior to harvesting cells. Everolimus: 500nM; α-Vinculin was used as a loading control.

We next sought to determine whether the upregulation of MYC expression observed in eveR derivatives was of functional consequence in the everolimus-resistant setting. To address this, two sequence-specific siRNAs to MYC were employed (Figure 3A). Importantly, knockdown of MYC resulted in partial resensitization of MCF7-eveR lines to everolimus in a short term proliferation assay (Figure 3B). Sensitivity to everolimus was also restored in MCF7-LTED-eveR lines transfected with either *MYC* siRNA (Figure 3B). Furthermore, colony formation assays revealed a reduced ability of both eveR derivatives to form colonies when expressing either *MYC* siRNA in the presence of everolimus (Figure 3C). Intriguingly, the MCF7-LTED line showed increased growth inhibition when everolimus was combined with either *MYC* siRNA (Figure 3B). This suggests that the expression of MYC (whether pre-existing or in response to everolimus treatment) can play a role in determining sensitivity of LTED derivatives to everolimus.

To determine whether MYC was sufficient to cause resistance to everolimus we employed a tet-inducible vector to overexpress MYC in MCF7 parental lines. In the presence of doxycycline, MYC expression was upregulated in MCF7 cells (Figure 3D). We then exposed the cells to increasing concentrations of everolimus in the presence or absence of MYC expression (+/− doxycycline). Interestingly, MYC expression resulted in significant recovery of proliferation even at the highest concentrations of everolimus (Figure 3D). This was in comparison to cells expressing tet-inducible RFP, which were sensitive to everolimus regardless of exposure to doxycycline and the expression of RFP. These results suggest that MYC expression alone can confer resistance to everolimus in MCF7 cells. These data underscore the importance of the MYC upregulation observed in the eveR derivatives and demonstrate a direct connection between MYC expression and everolimus sensitivity.

### BRD4-mediated upregulation of MYC expression in the everolimus-resistant setting

**Figure 4:**
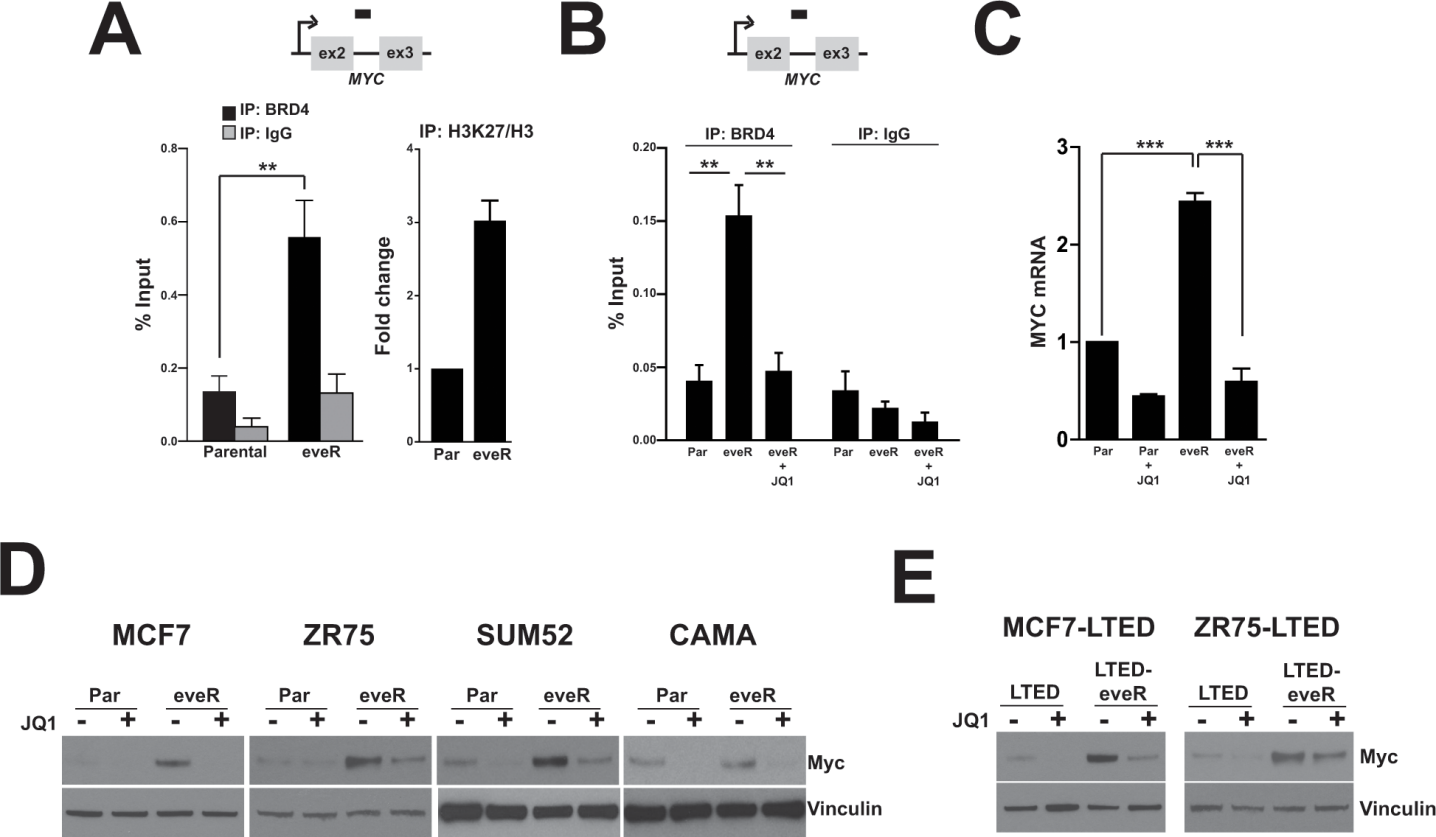
MYC is upregulated in the eveR setting in a BRD4-dependent manner A. Chromatin immunoprecipitation (ChIP) analyses of MCF7 Parental or MCF7-eveR. (left) ChIP experiments were performed with an antibody specific to BRD4 (Bethyl Labs). or IgG control. Bars represent the average % input as measured by real-time PCR analyses using primers specific for the *MYC* gene from four independent chromatin immunoprecipitations. Bars represent SEM. Student's *t*-test was performed to calculated statistical significance. **p-value=0.0082. (right) H3K27me3 histone ChIPs, % input was calculated from three independent ChIPs using an antibody specific for acetylated H3K27, and were then normalized to % input from total H3. Average fold change compared to parental is shown in the histogram with SEM. B. ChIP performed similar to (A) on MCF7 Parental cells (Par), MCF7-eveR cells (eveR) or MCF7-cells treated with 500nM JQ1 for 24h (eveR + JQ1). **Student's *t*-test was performed to calculated statistical significance. MCF7-eveR compared to MCF7 Parental *p*-value =0.0012; MCF7-eveR compared to MCF7-eveR + JQ1 *p*=0.0021. C. Real-time analyses in MCF7 Parental cells (Par), MCF7 cells treated with 500nM JQ1 for 24h (Par + JQ1), MCF7-eveR cells (eveR) or MCF7-eveR treated with 500nM JQ1 for 24h (eveR + JQ1) with MYC specific primers. Data is an average of three biological replicates and is represented as fold change over MCF7 Parental with SEM. MCF7-eveR compared to MCF7-Parental: ***p=8.9E=-05. MCF7 eveR compared to MCF7-eveR + JQ1: ***p=0.000341. D and E. Western blot analyses of indicated cell lines. Cells were treated with JQ1 (500nM, 24h). Cells were harvested post-treatment, lysed and extracted protein was immunoblotted for MYC. α-Vinculin was used as a loading control.

BRD4 (bromodomain-containing protein 4) is a transcriptional regulator that recognizes and binds acetylated histones and drives transcription of many genes, including *MYC*. While MYC regulation by BRD4 has been extensively studied in hematological malignancies, relatively little is known about this mechanism in solid tumors, particularly in breast cancer. A recent publication outlined a role for BRD-mediated transcription of MYC in tamoxifen-resistant breast cancers [20]. As mentioned above, the LTED setting mimics patient response to aromatase inhibition and tamoxifen treatment [10]. Our observations of *MYC* upregulation in the eveR setting and this novel connection between BRD regulation and tamoxifen resistance prompted us to investigate BRD4 as a potential regulator of MYC in the eveR lines. In order to determine whether MYC upregulation is mediated by enhanced BRD4 association with the *MYC* gene we performed chromatin immunoprecipitation (ChIP) analyses. We observed that eveR lines showed increase binding of BRD4 to the *MYC* gene (Figure 4A, left). We also performed ChIP using antibodies specific to acetylated H3K27, a mark of active transcription. We observed an acetylated H3K27 mark on the *MYC* gene in the MCF7-eveR line relative to MCF7 parental (Figure 4A, right). These data are consistent with the observation that *MYC* is upregulated at the transcript level in the eveR setting (Figure 2). Recently, the small molecule JQ1 was shown to inhibit BRD4-dependent transcription by preventing the binding of the BRD4 bromodomain to acetylated lysine residues [21]. To further test the specific regulation of *MYC* by BRD4 in the eveR setting we used JQ1 in our ChIP analyses. Treatment with JQ1 resulted in abrogation of BRD4-occupancy at the *MYC* gene in eveR lines (Figure 4B). This experiment was repeated with a second BRD4-specific antibody and obtained similar results (Supplemental Figure 2). In concordance with this, JQ1 treatment resulted in downregulation of MYC mRNA expression in the eveR lines (Figure 4C). In fact, JQ1 treatment abolished MYC upregulation in all eveR and LTED-eveR cell lines, suggesting that BRD4 is responsible for the increased MYC expression in these settings (Figure 4D and 4E). Interestingly, fractionation of MCF7 derivatives revealed that global association of BRD4 with chromatin does not change in the eveR lines (data not shown). In addition, neither MCF7 nor ZR75 eveR derivatives showed an increase in BRD4 mRNA expression (Supplemental Figure 3).

These results together suggest that specific BRD4 association with the MYC gene is enhanced in eveR lines and is responsible for governing high MYC expression in this context.

### Combination of everolimus and JQ1 leads to enhanced growth inhibition

**Figure 5:**
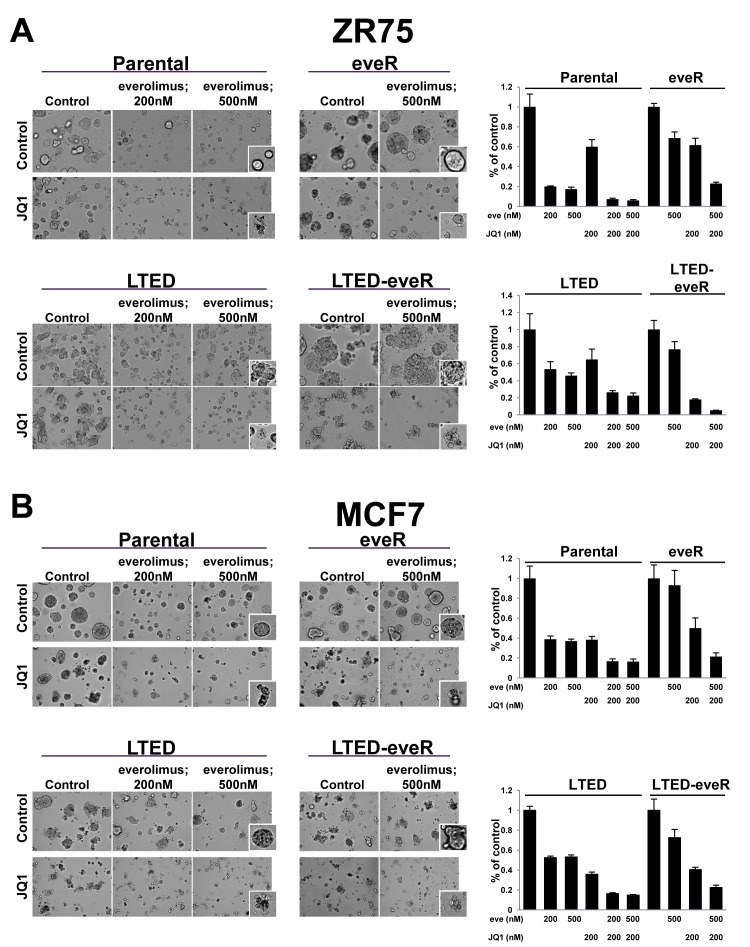
Combination effect observed with JQ1 and everolimus treatment A and B. (left) ZR75 (top) or MCF7 (bottom) derivative cell lines were grown in three-dimensional Matrigel culture for six days in the presence of indicated compounds (JQ1; 200nM) and representative photomicrographs were taken using a 20X objective. (right) Proliferation of 3D structures was measured by CellTiterGlo six days post-treatment. An average percent of control is depicted in the histograms with standard deviation. eve: everolimus

Given the aforementioned role of MYC as a driver of everolimus resistance, and as a target for BRD4 regulation in this setting, we hypothesized that treatment with JQ1, a small molecule inhibitor of BRD4, could be combined with everolimus to increase growth inhibition. The 3D Matrigel assay allows us to examine changes in growth while taking into consideration ECM and basement membrane interactions that more closely mimic growth *in vivo* [16]. Therefore, this assay was used to examine responses to combinations of everolimus and JQ1 (Figure 5). MCF7 and ZR75 derivatives were plated in Matrigel and their sensitivity to everolimus, JQ1 and the combination were examined. ZR75 Parental cells were relatively sensitive to everolimus alone at both concentrations and as previously shown (Figure 1 and 5A). However, the treatment with everolimus did not result in cell death. Rather, single cells remained intact and relatively viable in culture. Treatment with JQ1 in combination, however, resulted in an even further decrease in cell number (Figure 5A, right). This was consistent with the increased disintegration of cells depicted in the everolimus + JQ1 treatment group, suggesting the addition of JQ1 caused cell death. As expected, everolimus treatment of the ZR75-eveR line resulted in minimal sensitivity to the high concentration of everolimus, however when combined with JQ1, the lines were resensitized to everolimus and the combination resulted in greater attenuation of proliferation (Figure 5A). In fact, JQ1 resensitized the eveR lines to everolimus to a level comparable to that of the parental cells treated with everolimus alone. The ZR75-LTED lines exhibited less sensitivity to everolimus alone than the parental lines as seen previously (Figure 1 and Figure 5A). Combinations of everolimus and JQ1 in the LTED cells significantly decreased growth compared with either agent alone. Similar to parental cells, the combination also resulted in increased cell disintegration, indicative of cell death (Figure 5). Interestingly, the ZR75-LTED-eveR line was very sensitive to JQ1, even in the absence of everolimus. The combination of both drugs significantly reduced proliferation and greatly induced cell disintegration in the ZR75-LTED-eveR line (Figure 5). Similar results were seen with MCF7 cells (Figure 5B).

In SUM52 and CAMA cell lines, we performed a short term proliferation assay with everolimus, JQ1 and the combination (Supplemental Figure 4A). In both lines, addition of JQ1 to everolimus-treated cells resulted in increased growth inhibition compared with everolimus treatment alone. Furthermore, a comprehensive 6×6 combination grid of ZR75 derivatives in 2D treated with both compounds showed an increased growth inhibitory effect when JQ1 was combined with everolimus (Supplemental Figure 4B). Interestingly, ZR75-LTED-eveR were resensitized to everolimus to greater extent in the presence of JQ1 than the ZR75-eveR cell line. These results suggest that a BRD4-mediated mechanism of driving resistance can be targeted in the everolimus-resistant setting, as well as in combination with acute treatments of everolimus to increase efficacy.

### Inhibition of mTOR and BRD4 results in greater efficacy in an *in vivo* xenograft model

**Figure 6:**
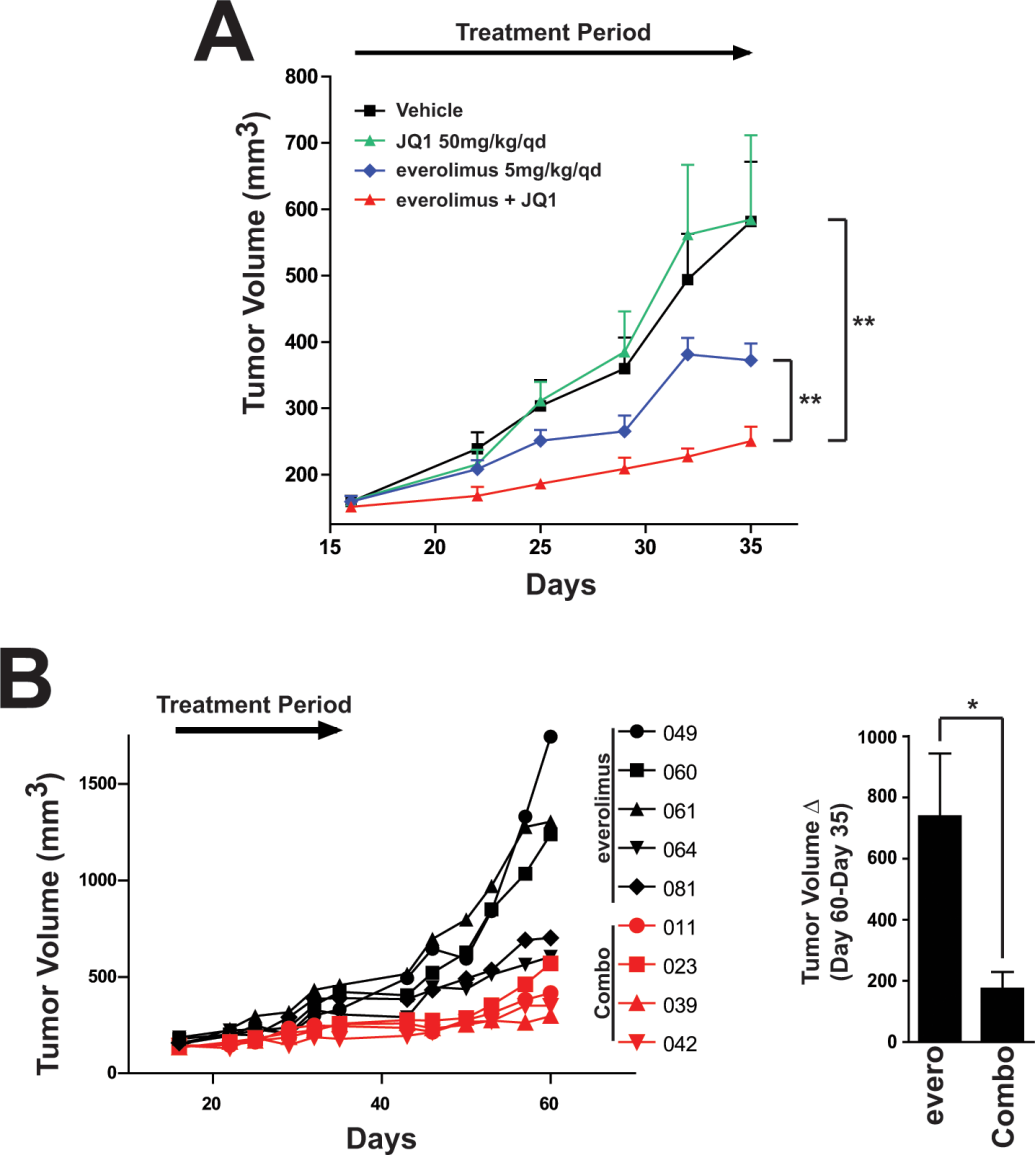
Combination of everolimus and JQ1 results in increased tumor growth inhibition in an *in vivo* MCF7 xenograft model A. Tumor volume measurements in the MCF7 *in vivo* tumor xenograft. Mice were supplemented with estrogen pellets (0.18mg/90-day release) and were treated with either vehicle, everolimus, JQ1 or the combination as indicated (n=8/9 for each group).** p<0.01. B. Individual tumor measurements from A. (left) Individual animals were measured for regrowth post-treatment with everolimus or the combination of everolimus and JQ1 (Combo). Average tumor volume change post-treatment from Day 35 to Day 60. Bars represent SEM. *p<0.05, evero: regrowth in everolimus-treated samples. Combo: regrowth in everolimus + JQ1 samples.

Given the results in 3D, we hypothesized that we could achieve increased tumor growth inhibition of everolimus and JQ1 *in vivo*. We tested the combination in an MCF7 *in vivo* xenograft model. After the tumors reached ~150mm^3^, mice were treated with vehicle control, everolimus, JQ1 or the combination. Treatment with JQ1 showed no efficacy compared to the vehicle control (Figure 6A). Everolimus treatment alone resulted in only 50% tumor growth inhibition (TGI). While the tumor growth inhibition in response to everolimus was significantly different than the vehicle (*p*=0.005), we believe the partial sensitivity would not translate into a durable clinical response. Strikingly, while JQ1 has no effect alone, the addition of JQ1 to everolimus resulted in a statistically significant tumor growth inhibition compared to either agent alone (*p*=0.008 compared to everolimus alone and *p*=0.0017 compared to JQ1 alone, TGI= 75% relative to vehicle control). This suggests a combination benefit for these two agents in producing a near complete response in this *in vivo* model (Figure 6A). Furthermore, this suggests the need for additional therapies to treat the residual and potentially resistant population. Importantly, body weight loss did not exceed 20% in any of the treatment groups, suggesting the agents were well-tolerated (data not shown). To determine whether the addition of JQ1 to everolimus would alter regrowth capabilities post-treatment, we measured animals after treatment had ended in the everolimus alone and in the combination group. The combination-treated animals had severely impaired regrowth post-treatment compared to animals that had received everolimus alone (Figure 6B). In fact, the average change in tumor volume was statistically significant between groups (Figure 6B, right). In conclusion, treatment with everolimus *in vivo* results in greater *in vivo* efficacy when combined with JQ1 suggesting a benefit to adding a BRD4 inhibitor to an mTOR inhibitor treatment regimen.

## DISCUSSION

Here, we demonstrate that resistance to the mTORC1 inhibitor, everolimus, can be acquired through the upregulation of MYC mediated by the transcriptional regulator BRD4. We have generated everolimus-resistant derivatives of several ER+ breast cancer lines. Through transcriptional profiling, we identified a MYC signature that is upregulated in eveR cells. This corresponds to transcriptional upregulation of MYC itself. Through overexpression and knockdown experiments, we have found that MYC is both necessary and sufficient for resistance to everolimus. Indeed, previous literature has linked MYC to the mTOR pathway. In a MYC-driven model of lymphoma, 4EBP1 is hyperphosphorylated in an mTOR-dependent manner [22]. In addition, MYC can cause resistance to rapamycin in prostate epithelial cells through a mechanism which paradoxically involves upregulation of 4EBP1 [23]. However, our data are not consistent with either of these mechanisms, as neither expression nor phosphorylation of 4EBP1 increases with MYC upregulation (Figure 1 and data not shown). Interestingly, MYC is also known to regulate ribosomal biogenesis through transcription of ribosomal RNAs [24]. Additionally, MYC has been shown to control autophagy, another process regulated by mTOR [25]. These functions may explain the ability of MYC to bypass the requirement for mTORC1 activity in this context.

Recently, publications have outlined MYC as a target for regulation by bromodomain protein, BRD4 [26]. Specifically, BRD4 has been shown to associate with the *MYC* gene in hematological malignancies and drive MYC expression [26]. Given the ability of BRD4 to regulate transcription factors that are difficult to target, such as MYC, BRD4 has become an attractive therapeutic target. Published studies have shown that in MYC-dependent tumors, JQ1 treatment causes reduced proliferation and increased differentiation as a consequence of MYC downregulation [21, 26-27]. Furthermore, another BRD4 inhibitor similar in structure to JQ1, OTX015, is currently being used in phase I clinical trials for hematological malignancies [28]. These recent developments underscore the therapeutic potential of targeting bromodomain proteins. Interestingly, we demonstrate MYC upregulation as a consequence of mTOR inhibition which requires BRD4, a known regulator of MYC. The BRD4 inhibitor JQ1 completely blocks MYC upregulation after everolimus treatment. With the recent emergence of BRD4 as a pharmacological target, this finding raises the intriguing possibility of using BRD4 inhibitors in the clinic to overcome everolimus resistance. We tested this possibility and observed that JQ1 is highly effective in killing eveR cells and enhances the antitumor effects of everolimus *in vivo*. In further support of this hypothesis, BRD4 was recently identified as a potential target in tamoxifen-resistant breast cancer [20], but has not yet been linked to mTOR inhibitors. It remains an open question how mTOR inhibition leads to BRD4 activation on the MYC gene. We do not see any change in the levels of BRD4 mRNA or its global association with chromatin after mTOR inhibition. Additionally, we did not observe any change in the expression of MCL1 (data not shown), another validated BRD4 target in hematological malignancies [29]. This not only demonstrates the tissue specificity of BRD4 function, it indicates the feasibility of using BRD4 inhibitors and the ability of BRD4 to regulate target genes such as *MYC* will have to be evaluated in each tumor type.

The differential sensitivity to everolimus depending on the cell line and background (LTED versus parental) suggests molecular players might be different between the parental and LTED background. Previous reports have demonstrated an activation of MYC in the LTED setting and MYC as a prognostic indicator of anti-estrogen treatment [17]. Indeed, we have observed an increase in MYC expression in LTED lines compared to parental lines (Figure 2G and data not shown). It is possible that this increased level of MYC has primed the cells for resistance to everolimus. Our data demonstrating lower sensitivity to everolimus in the LTED setting versus the parental setting parallels this argument (Figure 1 and 5). Interestingly, we observed a more robust response to the combination of everolimus and JQ1 in the ZR75-LTED-eveR lines compared to the ZR75-eveR cell line (Figure 5 and Supplemental Figure 4). Given that MYC levels can determine sensitivity to anti-estrogens [17] and, as shown here, everolimus-resistance, it is plausible that the LTED-eveR lines rely on MYC to a greater extent. Therefore, abolishing MYC levels in this setting is more detrimental to the cells. These data emphasize the importance of generating resistance in the order seen in the clinic, as it may have an impact on downstream signaling cascades and potentially, therapeutic response to future compounds.

While there are caveats to generating resistance in cell lines, as different contexts can rely on a variety of mechanisms for growth and response to treatment, we are encouraged by the fact that all four cell lines and their LTED derivatives display a unified upregulation of MYC, as compared to other relevant proteins (4EBP1 and ER levels). Moreover, we believe these cell lines' varied molecular contexts mimic patient heterogeneity, highlighting further the importance of MYC upregulation as a consistent phenotype. Furthermore, we are confident in the potential combinatorial strategy demonstrated by our data in 3D Matrigel and *in vivo* studies; growth conditions that better recapitulate the patient setting [16]. It will be important to see if similar mechanisms emerge from the BOLERO-2 trial samples and/or other clinical data involving mTOR inhibitors.

In summary, we present evidence for a novel mechanism that places BRD4 and MYC in a pathway which can promote resistance to mTORC1 inhibition. Combined inhibition of mTOR and BRD4 overcomes resistance and may represent a clinically relevant opportunity to enhance the therapeutic benefit of mTOR inhibitors.

## MATERIALS AND METHODS

### Cell Culture

MCF7, ZR75, CAMA-1 (ATCC) and SUM52 (Asterand) and non-LTED eveR derivatives were cultured in RPMI (Gibco) with 10% FBS (Sigma), 1% L-glutamine and antibiotics. For cells purchased from ATCC, cell lines were validated using short tandem repeat (STR) profiling and data was compared to the ATCC database. All LTED and LTED-EveR lines were generated and cultured in phenol red-free media + 10% charcoal dextran-treated fetal bovine serum (Hyclone), 1% L-glutamine and antibiotics. Everolimus resistance was generated by subjecting the parental or LTED derivative of each cell line to increasing concentrations of everolimus up to 500nM and maintained at this concentration. Generation of everolimus resistant pools took four to nine months depending on the cell line. During assays, cells were washed out of everolimus during plating and treated the following day with indicated compounds. MYC siRNA experiments were performed using either siRNA #1 (Dharmacon, D-003282-14-0020) or siRNA#2 (LifeTech, #S9130) or a non-targeting siRNA control (Dharmacon). Lines were transfected at a final concentration of 5nM and the following day cells were plated for growth assays. pTRIPZ constructs were obtained from Open Biosystems and viral transduction was performed as per manufacturer's instructions with packaging plasmids from Open Biosystems.

### Colony formation assays

Cells were plated in at least duplicate at 5000 cells per well of a 6-well dish. Media was replaced every 3-4 days with drug where indicated. Colonies were allowed to form for about 3 weeks or until control colonies were sufficient to be visualized by eye. At this point, plates were rinsed, fixed with 4% paraformaldehyde, stained with 0.1% crystal violet, rinsed and dried. Plates were scanned and representative photos are depicted.

### Three-dimensional Matrigel assays

Phenol-red free growth factor reduced Matrigel (BD) were plated as previously described [16]. After plating, cells were incubated overnight before adding the indicated concentrations of drug. Phase-contrast photographs were taken using a 20X objective using an Olympus DP71 microscope and images were captured using Olympus DP-BSW software.

### Immunoblotting

Cells were harvested and lysed in either TPER buffer (ThermoFisher, #78510) or cell lysis buffer (Cell Signaling) with phosphatase/protease inhibitor cocktail (Cell Signaling). The following antibodies were purchased from Cell Signaling Technologies: anti-p4EBP1 (S65) (#9456), anti-p-S6 (#4730), and anti-MYC (#5605). The following antibodies were purchased from Sigma: anti-Vinculin (#V9131). The following antibody was purchased from Millipore (anti-ER, 04-820).

### Quantitative Real-time PCR analysis

Cells were harvested and RNA was extracted using the RNeasy Plus Kit (Qiagen). RNA was quantified using a nano-drop and 2ug of total RNA was used to generate cDNA (High Capacity cDNA Synthesis Kit, Life Technologies). Per 10uL qPCR reaction, 2.5uL of cDNA was used. Triplicate reactions were run for each primer and sample set. The CT values were first normalized to HPRT housekeeping gene control (dCT), and then normalized to control or DMSO-treated samples (ddCT) depending on the experiment. Fold change was calculated using the following formula: 2^−ddCT^

### CellTiterGlo Assays

Cells were plated in 96-well plates. As per manufacturer's instructions, equal volumes of CellTiterGlo (Promega) was mixed with the cells and allowed to shake for 10-15 minutes. Luminescence was measured using a Tecan at a 1000 second exposure. Cells were normalized to Day 0 control and % net growth was determined using the following formula: ((x-y)/(z-y)) x100=% net growth where x=reading of treated sample at end of study, y=average reading on Day 0, and z=reading of DMSO-treated sample at end of study. The concentration of DMSO did not exceed 0.03% for any experiment.

### Chromatin Immunoprecipitation

All ChIP experiments were performed as previously described with the following modifications: after crosslinking, cell pellets were lysed directly in nuclear lysis buffer with 1% SDS [30]. The following primer sequences were used for ChIP analyses: *MYC*-(Forward): gccacctccatgctgtgt, *MYC* (reverse): agaactcctcctttccagtgc in the following region: chr8:128,805,744-128,809,484 (3741bp) build HG19 (UCSC Genome Browser). The primers were designed using Roche UPL.

### RNAseq Statistics and Visualizations

After treatment, cell pellets were sent to Expression Analysis (www.expressionanalysis.com) for RNA isolation, cDNA library generation, Illumina HiSeq RNAseq at 12 million read depths, and generation of the read FASTQ files. RNAseq quantification with Bcbio-nextgen (https://github.com/chapmanb/bcbio-nextgen) and edgeR [31-32] differential expression analysis were performed as previously described [33]. BROAD GSEA was also performed as previously described, except TMM normalized log2 count gene collapsed data was used for eveR versus Parental, LTED versus Parental, LTED-eveR versus LTED analyses as well as only using the c2.all.v4.0.symbols.gmt signature file from GeneSigDB [33-35].

### *In vivo* experiments

Female Ncr-nude mice were obtained from Taconic Laboratories and housed in pathogen-free housing in individually ventilated cages (IVC) of Polysulfone (PSU) plastic (mm 213 W x 362 D x 185 H, Allentown, USA) with sterilized and dust-free bedding cobs, access to sterilized food and water ad libitum, under a light-dark cycle (14-hour circadian cycle of artificial light) and controlled room temperature and humidity. During the study, mice were supplemented with estrogen pellets (0.18mg/90-day release; Innovative Research for America). For implantation, animals were anesthetized and 1×10^6^ MCF7 cells were implanted in a total of 50uL (of 50% Matrigel (BD): 50% RPMI media) transdermally in the third mammary fat pad. Tumors were measured with vernier calipers, and volumes were calculated using the formula (L*W^2^)*0.52. When the tumors reached an average of 150mm^3^, the mice were randomized into treatment groups by tumor volume. Animals were treated with vehicle control, everolimus at 5mg/kg p.o. and/or JQ1 at 50mg/kg i.p. once per day for 3 weeks. In combination treatment, JQ1 and everolimus were administered at least 6hrs apart to minimize any chance of drug-drug interaction. The control group received both i.p vehicle and p.o vehicle given on the same schedule as the combination group. All procedures were performed in accordance with federal, state and Institutional guidelines in an AAALAC-accredited facility and were approved by the AstraZeneca Institutional Animal Care and Use Committee.

## SUPPLEMENTARY MATERIAL, FIGURES, TABLES



## References

[1] Anderson WF, Chatterjee N, Ershler WB, Brawley OW (2002). Estrogen receptor breast cancer phenotypes in the Surveillance, Epidemiology, and End Results database. Breast Cancer Res Treat.

[2] Allred DC, Brown P, Medina D (2004). The origins of estrogen receptor alpha-positive and estrogen receptor alpha-negative human breast cancer. Breast Cancer Res.

[3] Ignatiadis M, Sotiriou C (2013). Luminal breast cancer: from biology to treatment. Nat Rev Clin Oncol.

[4] Shang Y, Hu X, DiRenzo J, Lazar MA, Brown M (2000). Cofactor dynamics and sufficiency in estrogen receptor-regulated transcription. Cell.

[5] Demers LM (1994). Effects of Fadrozole (CGS 16949A) and Letrozole (CGS 20267) on the inhibition of aromatase activity in breast cancer patients. Breast Cancer Res Treat.

[6] Geisler J, King N, Anker G, Ornati G, Di Salle E, Lonning PE, Dowsett M (1998). *In vivo* inhibition of aromatization by exemestane, a novel irreversible aromatase inhibitor, in postmenopausal breast cancer patients. Clin Cancer Res.

[7] Geisler J, King N, Dowsett M, Ottestad L, Lundgren S, Walton P, Kormeset PO, Lonning PE (1996). Influence of anastrozole (Arimidex), a selective, non-steroidal aromatase inhibitor, on *in vivo* aromatisation and plasma oestrogen levels in postmenopausal women with breast cancer. Br J Cancer.

[8] Wakeling AE, Dukes M, Bowler J (1991). A potent specific pure antiestrogen with clinical potential. Cancer Res.

[9] Ziauddin MF, Hua D, Tang SC (2014). Emerging strategies to overcome resistance to endocrine therapy for breast cancer. Cancer Metastasis Rev.

[10] Miller TW, Hennessy BT, Gonzalez-Angulo AM, Fox EM, Mills GB, Chen H, Higham C, Garcia-Echeverria C, Shyr Y, Arteaga CL (2010). Hyperactivation of phosphatidylinositol-3 kinase promotes escape from hormone dependence in estrogen receptor-positive human breast cancer. J Clin Invest.

[11] Lebwohl D, Anak O, Sahmoud T, Klimovsky J, Elmroth I, Haas T, Posluszny J, Saletan S, Berg W (2013). Development of everolimus, a novel oral mTOR inhibitor, across a spectrum of diseases. Ann N Y Acad Sci.

[12] Baselga J, Campone M, Piccart M, Burris HA, Rugo HS, Sahmoud T, Noguchi S, Gnant M, Pritchard KI, Lebrun F, Beck JT, Ito Y, Yardley D, Deleu I, Perez A, Bachelot T (2012). Everolimus in postmenopausal hormone-receptor-positive advanced breast cancer. N Engl J Med.

[13] Yardley DA, Noguchi S, Pritchard KI, Burris HA, Baselga J, Gnant M, Hortobagyi GN, Campone M, Pistilli B, Piccart M, Melichar B, Petrakova K, Arena FP, Erdkamp F, Harb WA, Feng W (2013). Everolimus plus exemestane in postmenopausal patients with HR(+) breast cancer: BOLERO-2 final progression-free survival analysis. Adv Ther.

[14] Piccart M, Hortobagyi GN, Campone M, Pritchard KI, Lebrun F, Ito Y, Noguchi S, Perez A, Rugo HS, Deleu I, Burris HA, Provencher L, Neven P, Gnant M, Shtivelband M, Wu C (2014). Everolimus plus exemestane for hormone-receptor-positive, human epidermal growth factor receptor-2-negative advanced breast cancer: overall survival results from BOLERO-2. Ann Oncol.

[15] Osborne CK, Schiff R (2011). Mechanisms of endocrine resistance in breast cancer. Annu Rev Med.

[16] Debnath J, Brugge JS (2005). Modelling glandular epithelial cancers in three-dimensional cultures. Nat Rev Cancer.

[17] Miller TW, Balko JM, Ghazoui Z, Dunbier A, Anderson H, Dowsett M, Gonzalez-Angulo AM, Mills GB, Miller WR, Wu H, Shyr Y, Arteaga CL (2011). A gene expression signature from human breast cancer cells with acquired hormone independence identifies MYC as a mediator of antiestrogen resistance. Clin Cancer Res.

[18] Bild AH, Yao G, Chang JT, Wang Q, Potti A, Chasse D, Joshi MB, Harpole D, Lancaster JM, Berchuck A, Olson JA, Marks JR, Dressman HK, West M, Nevins JR (2006). Oncogenic pathway signatures in human cancers as a guide to targeted therapies. Nature.

[19] Coller HA, Grandori C, Tamayo P, Colbert T, Lander ES, Eisenman RN, Golub TR (2000). Expression analysis with oligonucleotide microarrays reveals that MYC regulates genes involved in growth, cell cycle, signaling, and adhesion. Proc Natl Acad Sci U S A.

[20] Feng Q, Zhang Z, Shea MJ, Creighton CJ, Coarfa C, Hilsenbeck SG, Lanz R, He B, Wang L, Fu X, Nardone A, Song Y, Bradner J, Mitsiades N, Mitsiades CS, Osborne CK (2014). An epigenomic approach to therapy for tamoxifen-resistant breast cancer. Cell Res.

[21] Filippakopoulos P, Qi J, Picaud S, Shen Y, Smith WB, Fedorov O, Morse EM, Keates T, Hickman TT, Felletar I, Philpott M, Munro S, McKeown MR, Wang Y, Christie AL, West N (2010). Selective inhibition of BET bromodomains. Nature.

[22] Pourdehnad M, Truitt ML, Siddiqi IN, Ducker GS, Shokat KM, Ruggero D (2013). Myc and mTOR converge on a common node in protein synthesis control that confers synthetic lethality in Myc-driven cancers. Proc Natl Acad Sci U S A.

[23] Balakumaran BS, Porrello A, Hsu DS, Glover W, Foye A, Leung JY, Sullivan BA, Hahn WC, Loda M, Febbo PG (2009). MYC activity mitigates response to rapamycin in prostate cancer through eukaryotic initiation factor 4E-binding protein 1-mediated inhibition of autophagy. Cancer Res.

[24] Arabi A, Wu S, Ridderstrale K, Bierhoff H, Shiue C, Fatyol K, Fahlen S, Hydbring P, Soderberg O, Grummt I, Larsson LG, Wright AP (2005). c-Myc associates with ribosomal DNA and activates RNA polymerase I transcription. Nat Cell Biol.

[25] Toh PP, Luo S, Menzies FM, Rasko T, Wanker EE, Rubinsztein DC (2013). Myc inhibition impairs autophagosome formation. Hum Mol Genet.

[26] Zuber J, Shi J, Wang E, Rappaport AR, Herrmann H, Sison EA, Magoon D, Qi J, Blatt K, Wunderlich M, Taylor MJ, Johns C, Chicas A, Mulloy JC, Kogan SC, Brown P (2011). RNAi screen identifies Brd4 as a therapeutic target in acute myeloid leukaemia. Nature.

[27] Tolani B, Gopalakrishnan R, Punj V, Matta H, Chaudhary PM (2014). Targeting Myc in KSHV-associated primary effusion lymphoma with BET bromodomain inhibitors. Oncogene.

[28] ODORE KR E, RIVEIRO E, BOURDEL F, HERAIT P, CVITKOVIC E, DOMBRET H and, LOKIEC F (2014).

[29] Loven J, Hoke HA, Lin CY, Lau A, Orlando DA, Vakoc CR, Bradner JE, Lee TI, Young RA (2013). Selective inhibition of tumor oncogenes by disruption of super-enhancers. Cell.

[30] Black JC, Manning AL, Van Rechem C, Kim J, Ladd B, Cho J, Pineda CM, Murphy N, Daniels DL, Montagna C, Lewis PW, Glass K, Allis CD, Dyson NJ, Getz G, Whetstine JR (2013). KDM4A lysine demethylase induces site-specific copy gain and rereplication of regions amplified in tumors. Cell.

[31] Robinson MD, Smyth GK (2008). Small-sample estimation of negative binomial dispersion, with applications to SAGE data. Biostatistics.

[32] Robinson MD, McCarthy DJ, Smyth GK (2010). edgeR: a Bioconductor package for differential expression analysis of digital gene expression data. Bioinformatics.

[33] Ezell SA, Mayo M, Bihani T, Tepsuporn S, Wang S, Passino M, Grosskurth SE, Collins M, Parmentier J, Reimer C, Byth KF (2014). Synergistic induction of apoptosis by combination of BTK and dual mTORC1/2 inhibitors in diffuse large B cell lymphoma. Oncotarget.

[34] Subramanian A, Tamayo P, Mootha VK, Mukherjee S, Ebert BL, Gillette MA, Paulovich A, Pomeroy SL, Golub TR, Lander ES, Mesirov JP (2005). Gene set enrichment analysis: a knowledge-based approach for interpreting genome-wide expression profiles. Proc Natl Acad Sci U S A.

[35] Culhane AC, Schroder MS, Sultana R, Picard SC, Martinelli EN, Kelly C, Haibe-Kains B, Kapushesky M, St Pierre AA, Flahive W, Picard KC, Gusenleitner D, Papenhausen G, O'Connor N, Correll M, Quackenbush J (2012). GeneSigDB: a manually curated database and resource for analysis of gene expression signatures. Nucleic Acids Res.

