# Supplementary material for: Resistance to everolimus driven by epigenetic regulation of MYC in ER+ breast cancers
Source: Oncotarget. 2014 Dec 11;6(4):2407–20. doi: 10.18632/oncotarget.2964 (PMC4385860; doi:10.18632/oncotarget.2964)
Supplement: Supplementary file 1 [file oncotarget-06-2407-s001.pdf]

Resistance to everolimus driven by epigenetic regulation of MYC in ER+ breast cancers

Supplementary Material

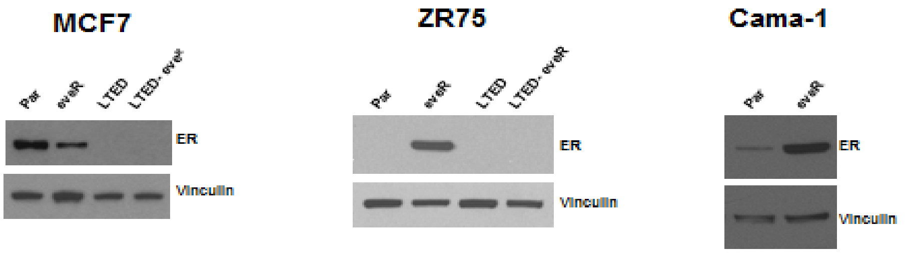

Supplemental Figure 1: Western blot analyses of indicated cell lines examining ER expression. α-Vinculin was used as a loading control.

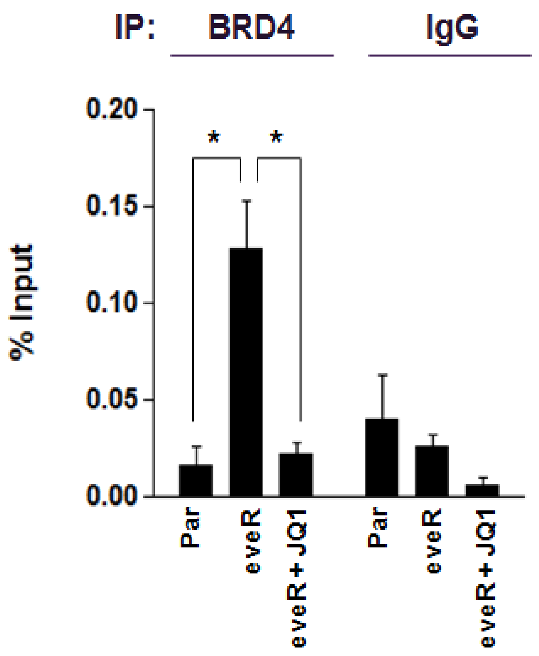

Supplemental Figure 2: ChIP performed on MCF7 Parental cells (Par), MCF7-eveR cells (eveR) or MCF7-cells treated with 500nM JQ1 for 24h (eveR + JQ1). ChIP experiments were performed with an antibody specific to BRD4 (Sigma) or IgG control. Bars represent the average percent input of three independent immunoprecipitations +/- SEM. Student's *t*-test was performed to calculated statistical significance. MCF7-eveR compared to MCF7 Parental p=0.0152; MCF7-eveR compared to MCF7-eveR + JQ1 p=0.0154.

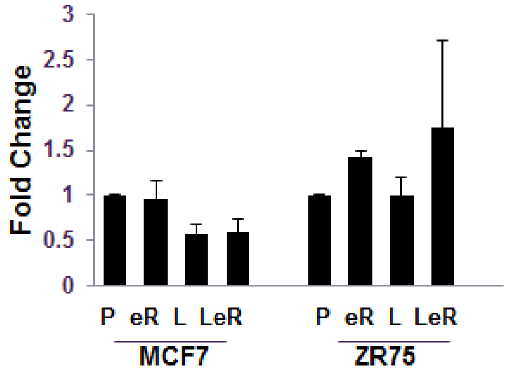

Supplemental Figure 3: Real-time analyses of BRD4 mRNA expression in the indicated lines (P: Parental, eR: eveR, L: LTED, LeR: LTED-eveR). Data is represented as fold change over parental controls with standard deviation in the histogram.

A

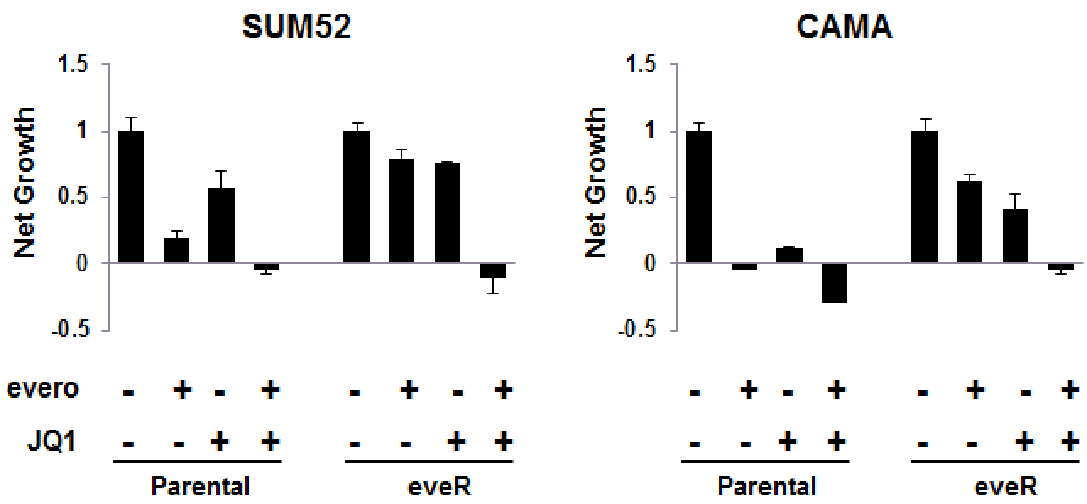

B

ZR75-eveR

JQ1 (uM) \ Everolimus (uM)	0	0.01	0.04	0.11	0.33	1.00
1.00	41	29	34	27	24	13
0.33	73	60	58	46	46	23
0.11	104	94	93	84	66	52
0.04	112	112	107	94	84	67
0.01	107	101	104	92	83	69
0	105	94	97	85	77	64

ZR75-LTED-eveR

JQ1 (uM) \ Everolimus (uM)	0.00	0.01	0.04	0.11	0.33	1.00
1.00	-15	-12	-8	-14	-12	-18
0.33	-2	-9	-8	0	-4	-11
0.11	29	12	18	13	17	-2
0.04	81	80	75	74	47	33
0.01	98	91	84	80	73	68
0	107	95	90	87	73	58

Everolimus (uM)

Supplemental Figure 4

A. Proliferation of indicated cells lines was measured after five days of the indicated treatments. Evero: 500nM everolimus, JQ1: 500nM. Average net growth is shown with standard deviation in the histograms.

B. ZR75 derivatives were plated in 96-well plates in triplicate in the absence of drug. One day after plating, everolimus and JQ1 were added at the indicated concentrations. Cells were incubated for five days before proliferation was measured by CellTiterGlo. Average percent net growth is depicted in the tables with a value of “0” representing 100% growth inhibition, a value of “100” representing 0% growth inhibition and a value of “-100” representing 100% cell death.

Supplemental Table 1:

MsigDB MYC gene signatures and the GSEA gene set enrichment for each expression comparison: eveR versus parental, LTED versus parental, as well as LTED-eveR and LTED. MYC signatures that are coordinately associated with the gene expression are in red text for up-regulated agreement and blue text for down-regulated agreement. Significant normalized enrichment scores are highlighted in yellow.

UP-REGULATED GENES EVER VERSUS PARENTAL & GSEA ENRICHED MYC SIGNATURE NAME	SIZE	ES	NES	NOM p-val	FDR q-val	FWER p-val	RANK AT MAX
SCHLOSSER_MYC_TARGETS_AND_SERUM_RESPONSE_DN	46	0.61273485	1.4800563	0.041353382	0.44489276	1	9632
SCHUHMACHER_MYC_TARGETS_UP	78	0.48110235	1.3225244	0.046728972	0.74082905	1	9626
WANG_NEOPLASTIC_TRANSFORMATION_BY_CCND1_MYC	21	0.5324874	1.0940754	0.30529594	0.9094968	1	3898
LEE_LIVER_CANCER_MYC_UP	54	0.4318744	1.0779904	0.2972973	0.9148604	1	7793
DANG_MYC_TARGETS_DN	31	0.4719338	1.072986	0.3286219	0.91263163	1	5298
LEE_LIVER_CANCER_MYC_TGFA_UP	61	0.41605508	1.064089	0.29910713	0.8980278	1	2374
KIM_MYC_AMPLIFICATION_TARGETS_UP	196	0.3425695	1.035287	0.2890625	0.9023585	1	8805
SCHLOSSER_MYC_TARGETS_REPRESSED_BY_SERUM	156	0.36190924	1.0326123	0.32608697	0.89373535	1	9974
SCHLOSSER_MYC_TARGETS_AND_SERUM_RESPONSE_UP	46	0.42069438	1.0248966	0.38321167	0.90530777	1	9886
COLLER_MYC_TARGETS_UP	25	0.4531283	0.9848087	0.4454023	0.9036821	1	9063
MENSSEN_MYC_TARGETS	51	0.38247973	0.9253916	0.5695067	0.95110404	1	9358
SANSOM_WNT_PATHWAY_REQUIRE_MYC	58	0.35162094	0.8847099	0.70689654	0.9913445	1	7606
ACOSTA_PROLIFERATION_INDEPENDENT_MYC_TARGETS_UP	78	0.29737964	0.79474425	0.9859155	1	1	9138
CEBALLOS_TARGETS_OF_TP53_AND_MYC_UP	21	0.3413045	0.7116354	0.9318885	1	1	2365
**DOWN-REGULATED GENES EVER VERSUS PARENTAL & GSEA ENRICHED MYC SIGNATURE NAME**	**SIZE**	**ES**	**NES**	**NOM p-val**	**FDR q-val**	**FWER p-val**	**RANK AT MAX**
LEE_LIVER_CANCER_MYC_TGFA_DN	62	-0.666958	-1.483026	0.010526316	0.54183185	1	6544
LEE_LIVER_CANCER_MYC_E2F1_DN	62	-0.61236376	-1.3655531	0.0265252	0.89933866	1	9734
SANSOM_APC_MYC_TARGETS	204	-0.4751587	-1.181894	0.094104305	0.87886804	1	15025
YU_MYC_TARGETS_UP	40	-0.5616763	-1.1728138	0.22366522	0.8640079	1	14988
SCHLOSSER_SERUM_RESPONSE_AUGMENTED_BY_MYC	102	-0.4957512	-1.1642625	0.16949153	0.87260526	1	15638
LEE_LIVER_CANCER_MYC_DN	61	-0.523163	-1.1632315	0.19363396	0.87085575	1	11360
MORI_EMU_MYC_LYMPHOMA_BY_ONSET_TIME_UP	104	-0.47658545	-1.1345936	0.20537898	0.8451941	1	15650
YU_MYC_TARGETS_DN	53	-0.5192721	-1.1289228	0.25268817	0.8371714	1	10642
MORI_EMU_MYC_LYMPHOMA_BY_ONSET_TIME_DN	17	-0.5974728	-1.0778778	0.35905045	0.8299678	1	5752
ODONNELL_TARGETS_OF_MYC_AND_TFRC_UP	81	-0.46127644	-1.0549	0.3753281	0.85037476	1	5046
KIM_MYC_AMPLIFICATION_TARGETS_DN	93	-0.44218323	-1.0307215	0.39874214	0.8666665	1	7436
BILD_MYC_ONCOGENIC_SIGNATURE	200	-0.40215272	-1.0156574	0.43348116	0.8750323	1	7360
SANSOM_APC_TARGETS_REQUIRE_MYC	199	-0.4000147	-1.007712	0.45936796	0.8799755	1	15064
DANG_REGULATED_BY_MYC_DN	250	-0.39608315	-1.0071012	0.44902387	0.88049436	1	6481
KIM_MYCN_AMPLIFICATION_TARGETS_UP	90	-0.42331654	-0.9938894	0.461837	0.88625324	1	6799
ODONNELL_TARGETS_OF_MYC_AND_TFRC_DN	45	-0.46315828	-0.9818207	0.5203366	0.89683133	1	15526
CEBALLOS_TARGETS_OF_TP53_AND_MYC_DN	38	-0.47251073	-0.9753772	0.5020464	0.90351236	1	6189
CAIRO_PML_TARGETS_BOUND_BY_MYC_UP	23	-0.5040204	-0.9702637	0.5167683	0.90877503	1	5
LEE_LIVER_CANCER_MYC_E2F1_UP	56	-0.42504013	-0.9264676	0.60053265	0.928335	1	5405
COWLING_MYCN_TARGETS	43	-0.4383698	-0.9194336	0.60714287	0.93564004	1	1441
PID_MYC_REPRESSPATHWAY	63	-0.41214272	-0.9188118	0.6251621	0.9350781	1	7284
ACOSTA_PROLIFERATION_INDEPENDENT_MYC_TARGETS_DN	112	-0.3711623	-0.88057774	0.75342464	0.94469804	1	7363
LASTOWSKA_COAMPLIFIED_WITH_MYCN	40	-0.41848034	-0.87396365	0.68049794	0.9482052	1	13592
BENPORATH_MYC_TARGETS_WITH_EBOX	227	-0.3425907	-0.863217	0.83387977	0.95061964	1	13220
SCHLOSSER_MYC_AND_SERUM_RESPONSE_SYNERGY	32	-0.42453608	-0.84315014	0.71468925	0.95465237	1	15717
ALFANO_MYC_TARGETS	234	-0.32885414	-0.83358806	0.8900979	0.9622769	1	7340
DANG_REGULATED_BY_MYC_UP	71	-0.357059	-0.8132023	0.8243065	0.9666048	1	14331
KIM_MYCN_AMPLIFICATION_TARGETS_DN	100	-0.34311253	-0.8042755	0.8817073	0.9690962	1	6412
FERNANDEZ_BOUND_BY_MYC	179	-0.3168694	-0.7957415	0.9187643	0.97169495	1	13062
ELLWOOD_MYC_TARGETS_DN	38	-0.37877387	-0.78128886	0.8333333	0.97674435	1	6794
PID_MYC_PATHWAY	25	-0.39973184	-0.77765197	0.80905235	0.9789333	1	13034
PID_MYC_ACTIVPATHWAY	79	-0.29322544	-0.6712784	0.9962121	1	1	14700
DANG_MYC_TARGETS_UP	141	-0.26933774	-0.6558347	1	1	1	15520
**UP-REGULATED GENES LTED VERSUS PARENTAL & GSEA ENRICHED MYC SIGNATURE NAME**	**SIZE**	**ES**	**NES**	**NOM p-val**	**FDR q-val**	**FWER p-val**	**RANK AT MAX**
LEE_LIVER_CANCER_MYC_E2F1_UP	56	0.5988493	1.3204461	0.04911839	0.75179386	1	5465
DANG_REGULATED_BY_MYC_DN	250	0.48340613	1.2303296	0.055075593	0.8305633	1	8970
YU_MYC_TARGETS_DN	53	0.5470489	1.1948729	0.15696202	0.8647524	1	6874
ODONNELL_TARGETS_OF_MYC_AND_TFRC_UP	81	0.48679143	1.1095848	0.2741546	0.9018371	1	7335
LEE_LIVER_CANCER_MYC_TGFA_UP	61	0.4946768	1.100696	0.31180125	0.8933966	1	4395
ALFANO_MYC_TARGETS	234	0.41502512	1.0411808	0.39423078	0.9190202	1	8444
SANSOM_WNT_PATHWAY_REQUIRE_MYC	58	0.46782526	1.0268328	0.43949044	0.9246236	1	4183
ACOSTA_PROLIFERATION_INDEPENDENT_MYC_TARGETS_DN	112	0.415217	0.99522233	0.48601398	0.94268763	1	8952
CEBALLOS_TARGETS_OF_TP53_AND_MYC_DN	38	0.47337136	0.9845885	0.4967062	0.9586646	1	7680
KIM_MYCL1_AMPLIFICATION_TARGETS_DN	19	0.53463036	0.98033667	0.52719665	0.9573213	1	4478
LASTOWSKA_COAMPLIFIED_WITH_MYCN	40	0.45139918	0.9483106	0.56078434	0.96208143	1	9210
KIM_MYC_AMPLIFICATION_TARGETS_DN	93	0.39834946	0.9292253	0.6461353	0.96596575	1	9262
CEBALLOS_TARGETS_OF_TP53_AND_MYC_UP	21	0.47655883	0.9002094	0.6325967	0.9731748	1	6913
LEE_LIVER_CANCER_MYC_DN	61	0.3932094	0.87179637	0.72166246	0.9791596	1	6653
KIM_MYCN_AMPLIFICATION_TARGETS_DN	100	0.36569288	0.8619843	0.76969695	0.98313034	1	9201
MORI_EMU_MYC_LYMPHOMA_BY_ONSET_TIME_UP	104	0.3663381	0.86184645	0.7423168	0.98298156	1	15388
BILD_MYC_ONCOGENIC_SIGNATURE	200	0.3454648	0.86120856	0.81857926	0.9817452	1	9599
DANG_MYC_TARGETS_UP	141	0.3457777	0.84045583	0.81127733	0.98159117	1	17266
DANG_MYC_TARGETS_DN	31	0.41531095	0.8375149	0.73698264	0.9828518	1	7563
SANSOM_APC_MYC_TARGETS	204	0.33226147	0.82882535	0.8849945	0.9852364	1	9302
CAIRO_PML_TARGETS_BOUND_BY_MYC_UP	23	0.43255118	0.8233946	0.7408907	0.98777324	1	16207
FERNANDEZ_BOUND_BY_MYC	179	0.3154093	0.7860247	0.92967033	0.99627626	1	9404

LEE_LIVER_CANCER_MYC_UP	54	0.35492423	0.7762709	0.85347044	0.9935759	1	9876
PID_MYC_REPRESSPATHWAY	63	0.3441623	0.7585641	0.89312977	0.9917899	1	7980
BENPORATH_MYC_TARGETS_WITH_EBOX	227	0.29283538	0.73978055	0.9913793	0.99039024	1	9593
ELLWOOD_MYC_TARGETS_DN	38	0.35425484	0.72987634	0.89986646	0.99079365	1	6270
SCHLOSSER_SERUM_RESPONSE_AUGMENTED_BY_MYC	102	0.30002517	0.708409	0.9811098	0.9971808	1	15190
PID_MYC_PATHWAY	25	0.36245927	0.6934992	0.9217274	0.998165	1	15681
ODONNELL_TARGETS_OF_MYC_AND_TFRC_DN	45	0.3233437	0.68364483	0.9490446	0.9990248	1	17708
**DOWN-REGULATED GENES LTED VERSUS PARENTAL & GSEA ENRICHED MYC SIGNATURE NAME**	**SIZE**	**ES**	**NES**	**NOM p-val**	**FDR q-val**	**FWER p-val**	**RANK AT MAX**
COLLER_MYC_TARGETS_UP	25	-0.5805363	-1.3070971	0.1042471	0.6791765	1	8481
MENSSEN_MYC_TARGETS	51	-0.47465116	-1.2055261	0.13658537	0.69120777	1	8299
KIM_MYCN_AMPLIFICATION_TARGETS_UP	90	-0.42371896	-1.180608	0.11111111	0.69145256	1	3891
WANG_NEOPLASTIC_TRANSFORMATION_BY_CCND1_MYC	21	-0.5509206	-1.165402	0.25538462	0.7095388	1	795
LEE_LIVER_CANCER_MYC_E2F1_DN	62	-0.43435583	-1.129476	0.20895523	0.72620064	1	6421
KIM_MYC_AMPLIFICATION_TARGETS_UP	196	-0.36113247	-1.1102613	0.15	0.73561835	1	3993
SCHLOSSER_MYC_TARGETS_AND_SERUM_RESPONSE_UP	46	-0.43192554	-1.0661347	0.30316743	0.75999445	1	3630
SCHUHMACHER_MYC_TARGETS_UP	78	-0.38056758	-1.0418952	0.30456853	0.79409355	1	8876
PID_MYC_ACTIVPATHWAY	79	-0.35031056	-0.9624044	0.54651165	0.86375475	1	7161
COWLING_MYCN_TARGETS	43	-0.38518903	-0.95919204	0.50427353	0.86902404	1	1919
LEE_LIVER_CANCER_MYC_TGFA_DN	62	-0.35197964	-0.9152889	0.6401869	0.90520793	1	6421
DANG_REGULATED_BY_MYC_UP	71	-0.33423167	-0.8932505	0.6930693	0.92341703	1	3397
ACOSTA_PROLIFERATION_INDEPENDENT_MYC_TARGETS_UP	78	-0.32794276	-0.8912759	0.735	0.9223271	1	8759
SCHLOSSER_MYC_TARGETS_AND_SERUM_RESPONSE_DN	46	-0.3609722	-0.88853455	0.6577778	0.92518914	1	8876
YU_MYC_TARGETS_UP	40	-0.3501658	-0.8529871	0.7393162	0.9586496	1	8833
SCHLOSSER_MYC_TARGETS_REPRESSED_BY_SERUM	156	-0.28785875	-0.8517611	0.95238096	0.9591085	1	8253
SANSOM_APC_TARGETS_REQUIRE_MYC	199	-0.26530448	-0.8173541	1	0.9836967	1	3889
SCHLOSSER_MYC_AND_SERUM_RESPONSE_SYNERGY	32	-0.34740144	-0.8010669	0.8515625	0.9819904	1	6962
MORI_EMU_MYC_LYMPHOMA_BY_ONSET_TIME_DN	17	-0.2966258	-0.6048476	0.993266	1	1	7106
**UP-REGULATED GENES LTEDEVER VERSUS LTED & GSEA ENRICHED MYC SIGNATURE NAME**	**SIZE**	**ES**	**NES**	**NOM p-val**	**FDR q-val**	**FWER p-val**	**RANK AT MAX**
LEE_LIVER_CANCER_MYC_E2F1_UP	56	0.41059935	1.0725232	0.28731343	0.7317954	1	3265
COLLER_MYC_TARGETS_UP	25	0.468108	1.0511543	0.34868422	0.74157554	1	3164
COWLING_MYCN_TARGETS	43	0.40959033	1.0424262	0.3381295	0.7446117	1	6652
SCHLOSSER_MYC_TARGETS_AND_SERUM_RESPONSE_DN	46	0.38864473	0.9984993	0.42532468	0.81475335	1	7932
WANG_NEOPLASTIC_TRANSFORMATION_BY_CCND1_MYC	21	0.43571097	0.953887	0.4936306	0.86788386	1	1138
SCHUHMACHER_MYC_TARGETS_UP	78	0.3407465	0.94143975	0.56578946	0.8839396	1	7631
PID_MYC_ACTIVPATHWAY	79	0.33520696	0.9269314	0.65975106	0.90471727	1	7763
ODONNELL_TARGETS_OF_MYC_AND_TFRC_UP	81	0.3341436	0.91459525	0.66390043	0.93245053	1	3642
MENSSEN_MYC_TARGETS	51	0.287149	0.75174123	0.99264705	1	1	3603
**DOWN-REGULATED GENES LTEDEVER VERSUS LTED & GSEA ENRICHED MYC SIGNATURE NAME**	**SIZE**	**ES**	**NES**	**NOM p-val**	**FDR q-val**	**FWER p-val**	**RANK AT MAX**
SANSOM_APC_MYC_TARGETS	204	-0.5736151	-1.5456637	0	0.25270975	1	18231
SCHLOSSER_MYC_TARGETS_REPRESSED_BY_SERUM	156	-0.58091956	-1.5219151	0	0.2755115	1	18665
ACOSTA_PROLIFERATION_INDEPENDENT_MYC_TARGETS_DN	112	-0.5732857	-1.4525794	0.003773585	0.35440233	1	11460
SCHLOSSER_SERUM_RESPONSE_AUGMENTED_BY_MYC	102	-0.56042933	-1.4168924	0.00996264	0.41601163	1	17794
LEE_LIVER_CANCER_MYC_TGFA_DN	62	-0.52416587	-1.2403038	0.1014885	0.506423	1	13408
KIM_MYCL1_AMPLIFICATION_TARGETS_DN	19	-0.6449213	-1.2294837	0.18545994	0.5122732	1	19085
HAHTOLA_MYCOSIS_FUNGOIDES_SKIN_UP	175	-0.4502357	-1.1968842	0.09489917	0.5489824	1	15914
SANSOM_APC_TARGETS_REQUIRE_MYC	199	-0.4411113	-1.183818	0.08986175	0.55761707	1	18165
YU_MYC_TARGETS_DN	53	-0.48408964	-1.1229658	0.2776243	0.63786614	1	10533
DANG_MYC_TARGETS_DN	31	-0.5204182	-1.1062152	0.31062672	0.6597855	1	13679
YU_MYC_TARGETS_UP	40	-0.50282943	-1.1037719	0.30822873	0.66119015	1	18093
KIM_MYCN_AMPLIFICATION_TARGETS_DN	100	-0.42231843	-1.0592542	0.3400251	0.733123	1	12830
DANG_MYC_TARGETS_UP	141	-0.4058957	-1.0518763	0.34093636	0.74565053	1	18740
MORI_EMU_MYC_LYMPHOMA_BY_ONSET_TIME_DN	17	-0.5424954	-1.0266726	0.4383358	0.7748807	1	10632
SCHLOSSER_MYC_AND_SERUM_RESPONSE_SYNERGY	32	-0.48632595	-1.0248505	0.4224138	0.7735273	1	17425
ALFANO_MYC_TARGETS	234	-0.37421536	-1.0225492	0.4140715	0.77562237	1	16496
BENPORATH_MYC_TARGETS_WITH_EBOX	227	-0.37616086	-1.0153018	0.41724136	0.78648883	1	18255
LEE_LIVER_CANCER_MYC_TGFA_UP	61	-0.42911613	-1.0068561	0.44065484	0.7929807	1	10878
MORI_EMU_MYC_LYMPHOMA_BY_ONSET_TIME_UP	104	-0.3926065	-0.9931243	0.48557088	0.8093733	1	18313
FERNANDEZ_BOUND_BY_MYC	179	-0.37159467	-0.9854701	0.5200472	0.8184115	1	17380
ODONNELL_TARGETS_OF_MYC_AND_TFRC_DN	45	-0.43635643	-0.9825428	0.47245178	0.8220247	1	18508
DANG_REGULATED_BY_MYC_DN	250	-0.3558622	-0.9672046	0.58305085	0.8360525	1	14372
LEE_LIVER_CANCER_MYC_E2F1_DN	62	-0.40829033	-0.9590052	0.538874	0.8466799	1	9913
DANG_REGULATED_BY_MYC_UP	71	-0.39901346	-0.9555575	0.5562914	0.8479631	1	17607
KIM_MYC_AMPLIFICATION_TARGETS_DN	93	-0.3886161	-0.95243806	0.57106596	0.8476713	1	13748
CEBALLOS_TARGETS_OF_TP53_AND_MYC_DN	38	-0.4205585	-0.9255399	0.5934844	0.8746611	1	13452
KIM_MYCN_AMPLIFICATION_TARGETS_UP	90	-0.36243752	-0.8890221	0.6975547	0.9092199	1	11968
ELLWOOD_MYC_TARGETS_DN	38	-0.3856066	-0.84756404	0.7277937	0.94524384	1	14033
BILD_MYC_ONCOGENIC_SIGNATURE	200	-0.30666205	-0.82528794	0.9162844	0.95439523	1	15072
CAIRO_PML_TARGETS_BOUND_BY_MYC_UP	23	-0.41308227	-0.8194721	0.74545455	0.9611689	1	17405
PID_MYC_REPRESSPATHWAY	63	-0.3449593	-0.8192705	0.8319892	0.96078837	1	16981
LEE_LIVER_CANCER_MYC_DN	61	-0.34589827	-0.81734186	0.8165997	0.96312207	1	12959
CEBALLOS_TARGETS_OF_TP53_AND_MYC_UP	21	-0.40947148	-0.81434286	0.7454819	0.9639744	1	18099
KIM_MYC_AMPLIFICATION_TARGETS_UP	196	-0.29416567	-0.78390896	0.9596774	0.9813256	1	15150
LEE_LIVER_CANCER_MYC_UP	54	-0.32391366	-0.74924487	0.92789114	0.9918167	1	14435
SANSOM_WNT_PATHWAY_REQUIRE_MYC	58	-0.3133619	-0.7324413	0.9498645	0.99514383	1	15541
PID_MYC_PATHWAY	25	-0.3549877	-0.7304552	0.89196676	0.9945192	1	17416
ACOSTA_PROLIFERATION_INDEPENDENT_MYC_TARGETS_UP	78	-0.2957563	-0.7189551	0.9755155	0.99486995	1	18437
SCHLOSSER_MYC_TARGETS_AND_SERUM_RESPONSE_UP	46	-0.31666583	-0.7174099	0.9436039	0.99580365	1	18396
LASTOWSKA_COAMPLIFIED_WITH_MYCN	40	-0.31374827	-0.6936123	0.97890294	0.99922687	1	12788
